# Modelling the Contribution of Metacognitions, Impulsiveness, and Thought Suppression to Behavioural Addictions in Adolescents

**DOI:** 10.3390/ijerph18073820

**Published:** 2021-04-06

**Authors:** Yaniv Efrati, Daniel C. Kolubinski, Claudia Marino, Marcantonio M. Spada

**Affiliations:** 1 Faculty of Education and Society and Culture, Beit-Berl College, Kfar Sava 4490500, Israel; ypefrati@gmail.com; 2 Division of Psychology, School of Applied Sciences, London South Bank University, London SE1 0AA, UK; kolubid2@lsbu.ac.uk (D.C.K.); claudia.marino@unipd.it (C.M.); 3 Dipartimento di Psicologia dello Sviluppo e della Socializzazione, Università degli Studi di Padova, 35131 Padova, Italy

**Keywords:** adolescents, compulsive sexual behavior disorder, impulsiveness, internet gaming disorder, metacognitions, problematic social networks use, thought suppression

## Abstract

The most common behavioral addictions in adolescents are Internet Gaming Disorder (IGD), Compulsive Sexual Behavior Disorder (CSBD), and Problematic Social Networks Use (PSNU). In the present study, we investigated whether thought suppression and impulsiveness mediate the relationship between metacognitions and these three behavioral addictions (IGD, CSBD and PSNU). In Study 1 (*n* = 471), we examined whether online gaming thought suppression and impulsiveness mediate the relationship between metacognitions and IGD. In Study 2 (*n* = 453), we examined whether sex thought suppression and impulsiveness mediate the relationship between metacognitions and CSBD. In Study 3 (*n* = 1004), we examined whether social media thought suppression and impulsiveness mediate the relationship between metacognitions and PSNU. Results of path analysis indicated, across the three studies, the importance of both thought suppression and impulsiveness as mediators between metacognitions and the three behavioral addictions (IGD, CSBD and PSNU) being investigated. These findings provide an opportunity for therapists as well as educators to gain a better insight into the link between metacognitions, thought suppression, impulsiveness, and behavioral addictions as part of developmental behavior among adolescents.

## 1. Introduction

Behavioral addictions are a series of syndromes that are both identifiable and clinically significant, typically interfering with personal functioning as a consequence of engaging in compulsive rewarding behaviors that are not related to the use of addictive substances [1]. Typically, those suffering from behavioral addictions display chronic symptoms such as cravings, excessive tolerance, withdrawal, and impulsiveness. These can eventually have considerable adverse effects on an individual’s social, financial, and legal situation [2]. In adolescents, the most common behavioral addictions are Internet Gaming Disorder (IGD) [3], Compulsive Sexual Behavior Disorder (CSBD) [4,5] and Problematic Social Networks Use (PSNU) [6,7,8].

IGD refers to a problematic and habitual use of video games that involves compulsive behaviors, particularly the inability to control one’s playing habits. A person experiencing IGD will progressively prioritize playing video games over other activities and keep playing them even in the face of over one year of serious negative effects on their personal, familial, social, and occupational life [1]. Another addictive pattern, CSBD refers to an excessive and constant preoccupation with sexual thoughts, impulses, and behaviors. CSBD sufferers’ difficulties in controlling these symptoms frequently result in clinically significant problems, to the extent of compromising their functioning in one or more major areas of life (ICD-11; [9,10,11]). Although ICD-11 does not classify CSBD (classification number 6C72) as a behavioral addiction [12], several researchers do indeed identify it as such [13,14]. According to the literature, addictive behaviors can be defined as those involving actions that are performed often and consistently, and as a consequence of a strong urge, thereby impairing an individual’s ability to function in major aspects of life [15]. Addictions may also be characterized by a downward spiraling effect, psychological stress, or excitement or agitation before performing the addictive activity, and satisfaction, gratification, or relief during or after the activity. Over time, the effects that such behaviors have on addicted individuals usually become less gratifying, as people get accustomed to them. Negative reinforcement may also fuel some of these behaviors, which are often accompanied by strong urges [16]. There are several ways in which CSBD displays these tendencies: people affected by CSBD are likely to show signs of depression and anxiety when they refrain from sexual behavior (e.g., [17]), and they also find it difficult to cease or reduce their frequency of engaging in such behaviors [18].

PSNU refers to problems related to the drastic increase in use of online social networking [19] that share several characteristics relating to the development of addictive behaviors, including reinforcement schedules from new material posted online and the presence of classically conditioned cues, such as mobile notifications about the availability of new content. In addition, physiological arousal, and activation of appetitive pathways in response to social networks use, resemble those observed in other types of behavioral addiction, supporting the potential inclusion of problems related to social networks use in the category of non-substance addictions [20,21].

The worldwide prevalence among adolescents of IGD ranges between 7% and 15% [22], of CSBD between 12% and 18% [4], and of PSNU between 13% to 15% [7]. Several factors have been highlighted as important in understanding the etiology of behavioral addictions. In the current study, we focus on thought suppression and impulsiveness.

### 1.1. Impulsiveness and the Suppression of Thoughts in Addictive Behaviors

The conscious inhibition of thoughts or reasoning is a self-monitoring strategy characterized by efforts to manage psychological stress by maintaining a distance from unwelcome thoughts [23]. Thought suppression, understood as the attempt to ignore unwanted thoughts, is in fact one of the most common approaches for dealing with objectionable thoughts (e.g., [24]), especially if we feel that sharing them openly is not an option [25]. Ironically, the efforts made to suppress a given thought can result in a rebound effect, whereby that very thought becomes even more present in one’s mind [26,27]. Rebound effects have been associated with various addictive and addictive-like behaviors among adolescents and adults, such as CSBD [28,29], PSNU [19], IGD [30], alcoholism [31], smoking [32] and pathological betting [33], which gives an indication of the presence of a transdiagnostic role for thought suppression in addictive behaviors [34,35].

Impulsiveness can be considered a tendency to act and make decisions on a whim, without due consideration of the consequences [36,37]. Like attempts to suppress thoughts, impulsiveness has also been observed in tandem with various addictive and addictive-like behaviors among adolescents and adults including CSBD [29,38,39], PSNU [40], IGD [41], alcohol consumption [42], and compulsive eating [43]. According to a recent systematic literature review on the subject, impulsiveness may play a crucial role in substance and behavioral addictions [44]. With that being said, the association between impulsiveness and CSBD is less reliable as compared with IGD and PSNU, with several studies indicating no significant associations between the construct (e.g., [45,46]). In the current study, we, therefore hypothesized that thought suppression and impulsiveness would be positively correlated with IGD, and PSNU and with lesser extent with CSBD.

### 1.2. Metacognitions as Drivers of Thought Suppression and Impulsiveness

Although thought suppression and impulsiveness have been highlighted as important in the prediction of various behavioral addictions among adolescents and adults, limited studies have been undertaken on exploring the correlates of thought suppression and impulsiveness, especially among adolescents. In this research, we examine one key factor that might be associated with thought suppression and impulsiveness—metacognitions.

The term ‘metacognition’ refers to stable knowledge about one’s own cognitive system as well as strategies employed to regulate cognition and the awareness of the current state of cognition [47]. According to Wells and Matthews’ metacognitive model of psychological distress [47,48] metacognitions (beliefs about cognition) drive the activation of maladaptive coping strategies (rumination, worry, increased attention to threat and thought suppression) that exacerbate negative affect. This, in turn, increases the likelihood of engaging in addictive behaviors as an escapism and ‘last resort’ for achieving cognitive-affective self-regulation [35,49].

Cartwright-Hatton and Wells [50] developed the Metacognitions Questionnaire (MCQ; [50]) and the Metacognitions Questionnaire 30 (MCQ-30; [51]) aimed at assessing metacognitions. The MCQ and MCQ-30 consist of five factors: (i) positive beliefs about worry (e.g., “If I worry I will be solve the problem”); (ii) negative beliefs about thoughts concerning danger and uncontrollability (e.g., “My thoughts are out of control and may harm me”); (iii) cognitive confidence (e.g., “I don’t trust my judgement”); (iv) beliefs about the need to control thoughts (e.g., “I need to control my thoughts at all times”; and (v) cognitive self-consciousness (e.g., “I play close attention to how my mind works”).

A large body of research suggests that metacognitions are implicated in all psychological problems (for a review, see [52]). A recent systematic review by Hamonniere and Varescon [53] has also identified that metacognitions are associated with addictive behaviors in adults. Research, for example, has found that all five dimensions of the MCQ/MCQ-30 are positively correlated with the severity of alcohol use, nicotine use, gambling, and problematic Internet use [54,55,56,57,58,59,60,61,62,63,64,65,66,67]. These studies also indicated that among the five metacognitions factors, cognitive confidence, and beliefs about the need to control thoughts emerged as the strongest predictors of severity of addictive behavior. More recently research has also indicated that metacognitions are associated with both problematic social networks use and IGD in adolescents [68,69].

In the current study, and in line with the metacognitive model of psychological distress, we hypothesized that metacognitions would be positively correlated with both thought suppression and impulsiveness. According to this model, metacognitions should induce negative forms of coping strategies (thought suppression) as well as to a greater likelihood of being impulsive because of the paradoxical effect of engaging in mental control strategies (thought suppression, worry, rumination) that are likely to increase the salience of unwanted thoughts. Research has supported this premise by indicating that negative metacognitions are indeed associated with impulsiveness but not with sensation seeking (e.g., [70]) and with the activation and maintenance of coping strategies such as thought suppression (see [53] for a systematic review).

### 1.3. Possible Confounding Effects for Religiosity and Gender in Behavioral Addictions

Aside from examining the proposed model in which metacognitions lead to thought suppression and impulsiveness, which in turn are associated with IGD, CSBD and PSNU, in the current research we also examine whether the model stands after controlling for two background measures that were found to play a key role in addictive behaviors—religiosity and gender. Religion is often seen as a buffer or vanguard against addictive behavior of diverse origin [71,72,73]. In some aspects of life, however, religious beliefs may promote an inner struggle that might sustain an addictive behavior [74]. According to the moral incongruence model of problematic pornography use [75,76], for example, there is an incongruence between religious adolescents’ natural sexual urges and the conservative principles endorsed by religious people, such as a rabbi, and literature, such as the Bible or the Talmud, that discourage or even condemn sexual thoughts and behaviors. In keeping with this incongruence, several studies have shown that religious adolescents have higher levels of CSBD as compared with secular adolescents (e.g., [28,77,78]). These studies, however, were all conducted on Jewish populations (although Christianity and Islam share common negative views regarding expressing openly sexual behaviors) and so future studies should examine the robustness of these findings in other religions. Of note, to date, the relationship between religiosity, IGD and PSNU use has not been examined among adolescents [79]. Only one study found an association between religiosity and online gaming behavior among young adults (ages 17–31; [80]), and also identified that religiosity was linked with lower levels of gaming in general and lower risk for excessive/addictive gaming.

Gender differences are also common in behavioral addictions. For example, research indicates that boys are exposed to pornography at earlier age than girls, consume more pornography and tend more often to self-define themselves as addicted to pornography (see [81,82] for a recent reviews). In addition, boys have higher attentional bias toward sexual cues and tend to have higher prevalence of CSBD (e.g., [5,29,83]. Similarly, studies on IGD have shown a higher prevalence in boys [84,85,86,87]. Most studies report that boys have a 2–3 times greater risk of IGD than girls [85,86], with 4.1% of men and 3.2% of women reported as problematic players [88]. Conversely, some studies suggest that girls prefer to use the Internet for communication and boys for game playing [89], and indeed PSNU has been shown to be more prevalent in girls than boys [90].

**Figure 1:**
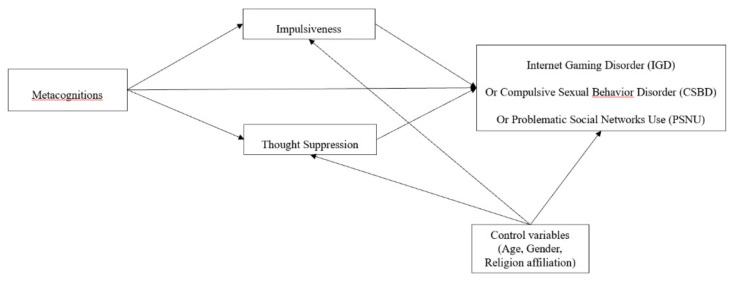
Hypothesized theoretical model.

The aim of the current study is to extend our understanding of the interlink between metacognitions, thought suppression, impulsiveness and three behavioral addictions (IGD, CSBD, and PSNU). We did this by testing a model where metacognitions predict both thought suppression and impulsiveness which in turn predict the behavioral addictions (see Figure 1). We did so while controlling for gender and religiosity. We hypothesized that: (1) metacognitions would be positively correlated with thought suppression and impulsiveness; and (2) thought suppression and impulsiveness would be positively correlated with IGD, CSBD, and PSNU. To do so, we conducted a series of three studies on a total of 1930 adolescents.

## 2. Study 1

We designed Study 1 to examine whether thought suppression and impulsiveness mediate the association between metacognitions (positive beliefs about worry, negative beliefs about thoughts concerning danger and uncontrollability, cognitive confidence, beliefs about the need to control thoughts, and cognitive self-consciousness) and IGD, such that higher metacognitions would be associated with higher thought suppression and impulsiveness, which in turn would be linked with greater severity of IGD.

### 2.1. Method

#### 2.1.1. Participants

For this study, a series of online questionnaires was completed by a sample of 474 teenagers across Israel (283 females; average age = 15.73 years [SD = 1.31; 14–18 years]). Out of the total sample, 23% classified themselves as secular, 25.5% classified themselves as traditional, 50.2% classified themselves as religious and 1.3% classified themselves as ultra-orthodox. Regarding religious affiliation, a dichotomous variable was created by combining ‘Secular’ and ‘Traditional’ under the variable ‘low religious affiliation’ and ‘Religious’ and ‘Ultra-Orthodox’ as ‘high religious affiliation’.

#### 2.1.2. Self-Report Measures

##### Metacognitions

The Metacognitions Questionnaire 30 (MCQ-30; [51]) is a 30-item self-report measure that assesses generic metacognitions in psychopathology using a 4-point Likert scale (1 = “Do not agree” and 5 = “Agree very much”). Five factors are assessed, which include: (a) positive beliefs about worry (POS); (b) negative beliefs about thoughts concerning danger and uncontrollability (NEG); (c) cognitive confidence (CC); (d) beliefs about the need to control thoughts (NC); and (e) cognitive self-consciousness (CSC). The higher the score, the higher the metacognitions level. The MCQ-30 has demonstrated good internal consistency and convergent validity and has acceptable test-retest reliability [51,62]. In this study, Cronbach’s alpha was 0.88.

##### Impulsiveness

The Barratt Impulsiveness Scale-11 (BIS-11; [91] translated to Hebrew by Glicksohn, Leshem and Aharoni [92]) is a 30-item self-report measure that assesses impulsiveness by means of a 4-point Likert scale (1 = Rarely/Never and 4 = Almost always/Always). It measures 3 factors: attentional impulsiveness (e.g., “It is difficult for me to stay engaged when I am trying to solve a logical problem”), motor impulsiveness (e.g., “I act on a whim without thinking about the consequences”) and non-planning impulsiveness (e.g., “I live in the present and don’t think much about the future”). Higher scores indicate higher levels of impulsiveness. Studies have shown that culture does not necessarily play a role in the definition of these three factors [93]. For example, Patton and colleagues [91]) have found consistent results with a BIS-11 total score ranging from 0.79 to 0.83 across different samples. In this study, Cronbach’s alpha was 0.80.

##### Thought Suppression

For this study, we adapted the Food Thought Suppression Inventory (FTSI; [94])) by translating the survey from English into Hebrew and then translating it into English again. The translators were instructed to replace each reference to thoughts about food with a reference to thoughts about online gaming. The Thought Suppression Inventory is a collection of 15 statements designed to assess a person’s tendency to suppress thoughts related to Internet gaming (e.g., “There are images about Internet gaming that come to mind that I cannot erase”) using a 5-point Likert scale (1 = Strongly disagree and 4 = Strongly agree). Higher scores indicate higher levels of thought suppression. The scores for Cronbach’s alpha in a population of women [94] and men [95] were found to be 0.96 and 0.95, respectively. In this study, Cronbach’s alpha was 0.94.

#### 2.1.3. Internet Gaming Disorder

The Internet Gaming Disorder Scale (IGDS9-SF; [96]), based on the nine IGD DSM-5 items [97] was used to assess the severity of IGD and its negative effects over a period of 1 year. The survey items were first translated from English into Hebrew by a translator who was fluent in both languages and then were back-translated into English by the author. Responses were rated on a 5-point scale (ranging from 1 = Never to 5 = Very often). Responses were averaged such that higher scores represent a higher internet gaming disorder severity. In this study, Cronbach’s alpha was 0.94.

### 2.2. Procedure

The Beit Berl Institutional Review Board (IRB) granted permission for the study to be conducted. Participants were recruited by postings on bulletin boards and online forums for volunteers for research on internet gaming among 14- to 18-year-old adolescents. The study questionnaires were uploaded to Qualtrics [98]. After adolescents responded and agreed to participate, their parents were contacted (by e-mail and/or phone) and were asked to review the questionnaires. If the parents approved, they were asked to sign an informed parental consent form and e-mail it to a research assistant. Following parental consent, a link for the online survey was sent to the adolescent, who was assured of the anonymity of the study. Participants were then asked to complete the survey at home, without anyone else present in the following order: metacognitions, impulsiveness, thought suppression, internet gaming disorder and socio-economic background measures. Before commencement of the study, each participant was requested to sign a statement of informed assent. After completion, the researchers contacted the students for a post-survey debriefing that took place online. At the very conclusion of the study, the adolescents were thanked for their participation.

### 2.3. Data Analysis

SPSS (version 25, IBM, New York, NY, USA; [99]) was used to calculate bivariate correlations among the variables. A series of Shapiro-Wilk normality tests indicated that all of the variables were non-normally distributed at the *p* < 0.001 level. Accordingly, all correlations were conducted using Spearman’s Rho (see Table 1). Gender was scored such that a positive correlation demonstrated higher scores among females and a negative correlation represented higher scores in males. Throughout these analyses, correlations between 0.1 and 0.3 were considered weak, correlations between 0.3 and 0.5 were considered moderate and correlations above 0.5 were considered strong.

Then, using path analyses, we tested the pattern of relationships indicated by our theoretical model (Figure 1). Specifically, we used the Lavaan package [100] of software R (Lavaan Project University of Gent, Gent, Belgium) [101] and a single observed score for each construct included in the model. We decided to use the Robust Maximum Likelihood method estimator (MLR; [102]) because several variables were non-normally distributed. To test for mediation, we used the Sobel test [103,104]. We considered the R^2^ value of each endogenous variable and the Total Coefficient of Determination (TCD; [105,106])) to assess whether the model was a good fit. In the tested models, internet gaming disorder was the outcome variable, impulsiveness and thought suppression were the mediators, and the five MCQ-30 metacognitions (i.e., positive beliefs about worry, negative beliefs about thoughts concerning danger and uncontrollability, cognitive confidence, beliefs about the need to control thoughts, and cognitive self-consciousness) were the independent variables, whereas age, gender, and religious affiliation (low vs. high) were included as control variables on the two mediators and the outcome (Figure 1).

**Figure 2:**
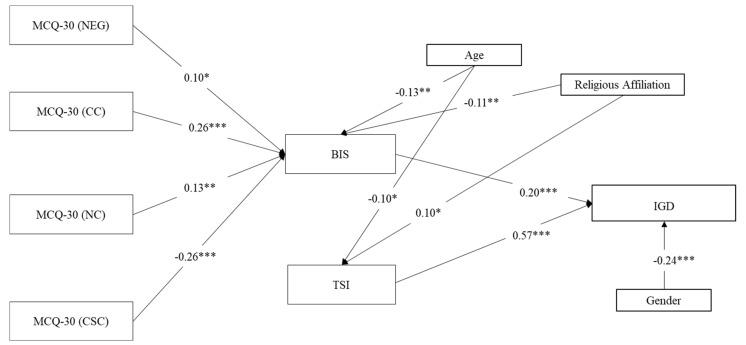
Results of the path analytical model with IGD as outcome variable. Notes: * *p* < 0.05, ** *p* < 0.001, *** *p* < 0.001; *n* = 471; Coefficients are standardized estimates; Religious Affiliation = (1 = Low, 2 = High); Gender: 1 = M, 2 = F; MCQ-30 (NEG) = Metacognitions Questionnaire-30 (Negative Beliefs about Thoughts concerning Uncontrollability and Danger); MCQ-30 (CC) = Metacognitions Questionnaire-30 (Cognitive Confidence); MCQ-30 (NC) = Metacognitions Questionnaire-30 (Beliefs about the Need to Control Thoughts); MCQ-30 (CSC) = Metacognitions Questionnaire-30 (Cognitive Self-Consciousness); BIS = Barret Impulsivity Scale; TSI = Thought Suppression Inventory; IGD = Internet Gaming Disorder.

We first tested the full model in order to assess which model was the most plausible. Subsequently, path coefficients not significant at the 5% level were removed step-by-step. For sake of brevity, Figure 2 shows the final model including significant path coefficient only.

### 2.4. Results

#### 2.4.1. Bivariate Correlations

The bivariate correlations between this study’s variables and the descriptive statistics for the variables can be found in Table 1. IGD was strongly correlated with thought suppression (*r_s_* = 0.67, *p* < 0.001) and had a weak correlation with impulsiveness (*r_s_* = 0.28, *p* < 0.001), gender (*r_s_* = −0.26, *p* < 0.001), cognitive confidence (*r_s_* = 0.22, *p* < 0.001), beliefs about the need to control thoughts (*r_s_* = 0.17, *p* < 0.001), negative beliefs about thoughts concerning uncontrollability and danger (*r_s_* = 0.15, *p* < 0.001), positive beliefs about worry (*r_s_* = 0.12, *p* < 0.001) and religiosity (*r_s_* = 0.10, *p* < 0.001).

#### 2.4.2. Path Analysis: Do Thought Suppression and Impulsiveness Mediate the Association between Metacognitions and IGD?

The model was run on a final sample of *n* = 471 adolescents (as three participants of the total sample of *n* = 474 did not complete one or more questionnaires). Negative beliefs about thoughts concerning uncontrollability and danger, cognitive confidence, and beliefs about the need to control thoughts were directly and positively associated with impulsiveness, whereas none of the five metacognitions was associated with thought suppression. However, a strong and positive correlation emerged between thought suppression and both IGD and impulsiveness. With respect to the control variables, religious affiliation was positively associated with thought suppression and negatively associated with impulsiveness, whereas gender was negatively associated with IGD, and age was negatively associated with both the mediators (impulsiveness and thought suppression) (Figure 2).

The results of the Sobel test with respect to indirect relationships did not support the mediating role of impulsiveness between negative beliefs about thoughts concerning uncontrollability and danger and IGD (*β* = 0.021, SE = 0.018, *z* = 1.929, *p* = 0.054) but did support the mediating role of impulsiveness between: (i) cognitive confidence and IGD (*β* = 0.053, SE = 0.024, *z* = 3.610, *p* < 0.001); (ii) beliefs about the need to control thoughts and IGD (*β* = 0.027, SE = 0.023, *z* = 2.381, *p* = 0.017); and (iii) cognitive self-consciousness and IGD (*β* = −0.052, SE = 0.025, *z* = −4.052, *p* < 0.001). No mediation paths were observed regarding thought suppression.

Considering the model fit, the model accounted for 42% of the variance of IGD, and 19% of the variance of one mediator (i.e., impulsiveness) variable. For the other mediator, a substantially lower variance was observed (i.e., thought suppression, 2%). Overall, it can be said that the model was an acceptable fit to analyze the data in light of the total amount of variance that it explained (total coefficient of determination, TCD = 0.28). In fact, a TCD of 0.28 corresponds to a correlation of *r* = 0.53, which can be reasonably described as a large effect size [107].

### 2.5. Discussion

Recently, research has indicated that metacognitions are linked with IGD among adolescents ([68]; also see [108] for a narrative review). Specifically, by assessing 515 Turkish adolescents aged 13.2 years, on average, the researchers found that metacognitions were linked with all the facets of IGD (the salience, mood modification, tolerance, withdrawal symptoms, conflict, and relapse of the disorder). In addition, metacognitions were associated with impulsiveness [70] and with the activation and maintenance of thought suppression [53]. In Study 1, we found support to the hypothesis that impulsiveness would mediate the association between metacognitions and IGD. Specifically, we found that lack of confidence in one’s mnemonic and attentional capabilities, beliefs about the need to control thoughts, and lack of cognitive self-consciousness, which reflects less monitoring of thinking processes, were associated with higher impulsiveness; higher impulsiveness, in turn, was linked with higher severity of IGD. These results are partially in keeping with [53] as well as Sun and colleagues’ [109] observations that beliefs about the need to control thoughts, and a lack of cognitive confidence are two of the metacognitions closely associated with addictive behaviors.

Conversely, although the Self-Regulatory Executive (S-REF, [48] 1994; CAS; [52,110]) models propose that metacognitions should be linked with the activation and maintenance of thought suppression, Study 1 did not reveal significant associations between metacognitions and thought suppression. One possible reason for the lack of association is that we did not assess adolescents’ overall tendency to suppress their thoughts but asked specifically about Internet gaming-related thought suppression. Because adolescents find Internet gaming as enjoyable and fulfilling, they might show less of a tendency to suppress thoughts related to excessive gaming. To examine this possibility and to explore our model in greater depth, we designed Study 2 in which we examined whether impulsiveness and thought suppression mediate the association between metacognitions and a behavioral addiction that more often incurs negative effects among adolescents and adults—CSBD.

## 3. Study 2

We designed Study 2 to examine whether thought suppression and impulsiveness mediate the association between metacognitions (positive beliefs about worry, negative beliefs about thoughts concerning uncontrollability and danger, cognitive confidence, beliefs about the need to control thoughts, and cognitive self-consciousness) and CSBD, such that higher metacognitions would be associated with higher thought suppression and impulsiveness, which in turn would be linked with greater severity of CSBD.

### 3.1. Method

#### 3.1.1. Participants

We recruited a sample of 453 teenagers (256 females; average age = 16.26 years [SD = 1.23; 14–18 years]) from across Israel and asked them to fill in a battery of questionnaires online. This was a different sample than Study 1. Out of the total sample, 39.1% classified themselves as secular, 25.4% classified themselves as traditional, 34.7% classified themselves as religious and 0.9% classified themselves as ultra-orthodox. As above, a dichotomous variable was created for religious affiliation by combining ‘Secular’ and ‘Traditional’ under the variable ‘low religious affiliation’ and ‘Religious’ and ‘Ultra-Orthodox’ as ‘high religious affiliation’.

#### 3.1.2. Self-Report Measures

##### Metacognitions

The Metacognitions Questionnaire 30 (MCQ-30; [51]) was used as in Study 1. In this study, Cronbach’s alpha was 0.88.

##### Impulsiveness

The Barratt Impulsiveness Scale-11 (BIS-11; [91] translated to Hebrew by Glicksohn, Leshem and Aharoni [92]) was used as in Study 1. In this study, Cronbach’s alpha was 0.82.

##### Thought Suppression

For this study, we adapted the Food Thought Suppression Inventory (FTSI; [94]) by translating the survey from English into Hebrew and then back-translating it into English. The translators were instructed to replace each reference to thoughts about food with a reference to thoughts about sex and sexuality. The Thought Suppression Inventory is a unidimensional collection of 15 statements that is meant to determine the tendency to suppress –thoughts related to sex (e.g., “There are images about sex that come to mind that I cannot erase”) using a 5-point Likert scale (1 = Strongly disagree and 4 = Strongly agree). Higher scores indicate higher levels of thought suppression. In this study, Cronbach’s alpha was 0.93.

##### Compulsive Sexual Behavior Disorder

The Individual-based Compulsive Sexual Behavior Scale (I-CSB; [111]) was designed to assess the severity of specific aspects of CSBD, including erotic fantasies, obsessive thoughts about sex, and the amount of time devoted to watching pornography. The I-CSB is a 24-item self-report measure assessing the following factors: Unwanted consequences (e.g., “I feel that my sexual fantasies hurt those around me”); lack of control (e.g., “I waste lots of time with my sexual fantasies”); negative affect (e.g., “I feel bad when I don’t manage to control my sexual urges”); and affect regulation (e.g., “I turn to sexual fantasies as a way to cope with my problems”). Using a 7-point Likert scale, participants were asked to rate the degree to which each statement is descriptive of their feelings (1 = Not at all and 7 = Very much). Higher scores indicate higher levels of compulsive sexual behavior. This self-report measure was successfully employed in previous research on non-clinical populations of adults and adolescents [112] and in clinical populations of Sexaholics Anonymous Twelve-Step program patients [111,113,114]. We calculated a total I-CSB score by finding the average score of the 24 items that composed the I-CSB.

### 3.2. Procedure

This was the same as in Study 1.

### 3.3. Data Analysis

**Figure 3:**
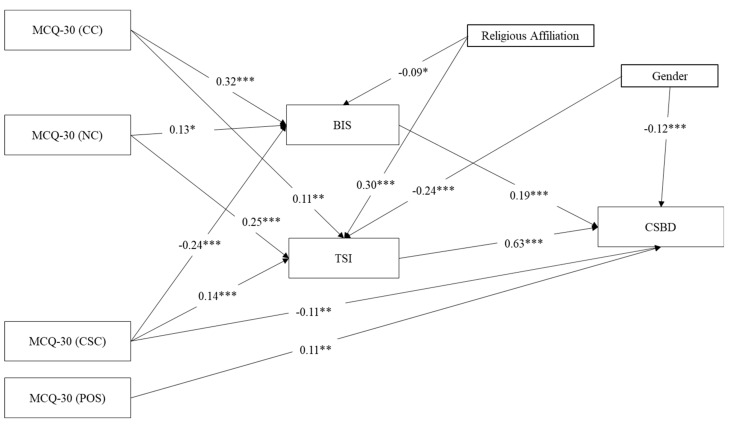
Results of the path analytical model with CSBD as outcome variable. Notes: * *p* < 0.05, ** *p* < 0.001, *** *p* < 0.001; *n* = 453; Coefficients are standardized estimates; Religious Affiliation = (1 = Low, 2 = High); Gender: 1 = M, 2 = F; MCQ-30 (POS) = Metacognitions Questionnaire-30 (Positive Beliefs about Worry); MCQ-30 (CC) = Metacognitions Questionnaire-30 (Cognitive Confidence); MCQ-30 (NC) = Metacognitions Questionnaire-30 (Beliefs about the Need to Control Thoughts); MCQ-30 (CSC) = Metacognitions Questionnaire-30 (Cognitive Self-Consciousness); BIS = Barret Impulsivity Scale; TSI = Thought Suppression Inventory; CSBD = Compulsive Sexual Behavior Disorder.

Using SPSS (version 25; IBM, New York, NY, USA [99]), we calculated bivariate correlations among the variables. A series of Shapiro-Wilk normality tests indicated that all of the variables were non-normally distributed at the *p* < 0.001 level. Accordingly, all correlations were conducted using Spearman’s Rho (see Table 1). Then, the pattern of relationships specified by our theoretical model (Figure 1) was tested using path analyses with the same parameters as in Study 1. Specifically, in the tested models, CSBD was the outcome variable, impulsiveness and thought suppression were the mediators, and the five MCQ-30 metacognitions (i.e., positive beliefs about worry, negative beliefs about thoughts concerning uncontrollability and danger, cognitive confidence, beliefs about the need to control thoughts, and cognitive self-consciousness) were the independent variables, whereas age, gender, and religious affiliation (low vs. high) were included as control variables on the two mediators and the outcome (Figure 1). We first tested the full model in order to assess which model was the most plausible. Subsequently, path coefficients not significant at the 5% level were removed step-by-step. For sake of brevity, Figure 3 shows the final model including significant path coefficient only.

### 3.4. Results

#### 3.4.1. Bivariate Correlations

CSBD was strongly correlated with thought suppression (*r_s_* = 0.68, *p* < 0.001) and had a weak correlation with impulsiveness (*r_s_* = 0.29, *p* < 0.001), gender (*r_s_* = −0.28, *p* < 0.001), beliefs about the need for control thoughts (*r_s_* = 0.28, *p* < 0.001), cognitive confidence (*r_s_* = 0.24, *p* < 0.001), positive beliefs about worry (*r_s_* = 0.15, *p* < 0.001), religiosity (*r_s_* = 0.15, *p* < 0.001) and negative beliefs about thoughts concerning uncontrollability and danger (*r_s_* = 0.14, *p* < 0.001).

#### 3.4.2. Path Analysis: Do Thought Suppression and Impulsiveness Mediate the Association between Metacognitions and CSBD?

The model was run on a sample of *n* = 453 adolescents (see Figure 3). Positive beliefs about worry were directly and positively associated with CSBD (although the association was not strong), while cognitive self-consciousness was associated directly and negatively with CSBD. Moreover, cognitive confidence and beliefs about the need to control thoughts were positively associated with impulsiveness and thought suppression, which, in turn were both positively associated with CSBD, with strongest association observed between thought suppression and CSBD. As regard the control variables, religious affiliation was positively associated with thought suppression and negatively associated with impulsiveness, whereas gender was negatively associated with both thought suppression and CSBD.

As far as indirect relationships are concerned, the results of the Sobel test highlighted the mediating role of impulsiveness between: (i) cognitive confidence and CSBD (*β* = 0.061, SE = 0.106, *z* = 4.066, *p* < 0.001); (ii) beliefs about the need to control thoughts and CSBD (*β* = 0.024, SE = 0.098, *z* = 2.023, *p* = 0.043); and cognitive self-consciousness and CSBD (*β* = −0.045, SE = 0.094, *z* = −3.412, *p* = 0.001). Moreover, results supported the mediating role of thought suppression between: (i) cognitive confidence and CSBD (*β* = 0.070, SE = 0.187, *z* = 2.623, *p* = 0.009); (ii) beliefs about the need to control thoughts and CSBD (*β* = 0.015, SE = 0.118, *z* = 2.042, *p* = 0.041); and (iii) cognitive self-consciousness and CSBD (*β* = −0.028, SE = 0.113, *z* = −3.446, *p* = 0.001).

As far as model fit is concerned, the model accounted for 49% of the variance of CSBD, and 30% of the variance of thought suppression. For impulsiveness, we observed a lower variance (17%), which is nonetheless significant. Overall, it can be said that the model was an acceptable fit to analyze the data in light of the total amount of variance that it explained (total coefficient of determination, TCD = 0.46). In fact, a TCD of 0.46 corresponds to a correlation of *r* = 0.68, which can be reasonably described as a large effect size [107].

### 3.5. Discussion

Study 2, to the best of our knowledge, is the first to examine the links between metacognitions and CSBD among adolescents, although research has found that early maladaptive schemas (which relate to distorted cognitions) are strong predictors of CSBD [114,115]. Here, we found that positive beliefs about worry were directly and positively associated with CSBD. Positive beliefs about worry are key n the activation of various forms of coping such as thought suppression in the presence of distressing triggers (e.g., upsetting thoughts, emotions, sensations). Such activation often backfires and leads to an escalation of negative affect [110] and, here, to a greater severity of CSBD.

In addition, the path model showed that lower cognitive self-consciousness, which reflects less monitoring of thinking processes, is also directly linked with a greater severity of CSBD. Given that one core facet of CSBD is the absence of behavioral control—persistent and uncontrolled engaging in behaviors, fantasies, and desires related to sex, accompanied by considerable fruitless efforts to reduce said compulsive sexual behaviors—it makes sense that the tendency to monitor fewer thinking processes will facilitate lack of sexual-related behavioral control and thus greater severity of CSBD.

Moreover, as in Study 1, similar mediation paths were revealed regarding impulsiveness. Specifically, we found that lack of confidence in one’s mnemonic and attentional capabilities, lack of belief about the need to control thoughts, and lack of cognitive self-consciousness were associated with higher impulsiveness; higher impulsiveness, in turn, was linked with higher severity of CSBD. Unlike Study 1, the same metacognitions were also significantly mediated by thought suppression: lack of confidence in one’s mnemonic and attentional capabilities, beliefs about the need to control thoughts, and lack of cognitive self-consciousness were associated with higher thought suppression; higher sex-related thought suppression was linked, in turn, with higher severity of CSBD. As suspected, given that CSBD incurs significant levels of negative affect, adolescents may try to suppress thoughts related to sex and sexuality and so, thought suppression may play a pivotal role in mediating the associations between metacognitions and CSBD.

Although Studies 1 and 2 supported our model regarding the role of metacognitions as well as impulsiveness (and to some extent thought suppression) in predicting behavioral addictions among adolescents, they explored addictive behaviors that are significantly more common among boys—IGD and CSBD (e.g., [85,86]). We designed Study 3 to further test our model regarding behavioral addictions by focusing on a behavioral addiction that is more prevalent among girls [90])—PSNU.

## 4. Study 3

We designed Study 3 to examine whether thought suppression and impulsiveness mediate the association between metacognitions (positive beliefs about worry, negative beliefs about thoughts concerning uncontrollability and danger, cognitive confidence, beliefs about the need to control thoughts, and cognitive self-consciousness) and PSNU, such that higher metacognitions would be associated with higher thought suppression and impulsiveness, which in turn would be linked with greater severity of PSNU.

### 4.1. Method

#### 4.1.1. Participants

For this study, we recruited a third sample (different from studies 1 and 2) of 1003 adolescents (621 females; average age = 16.04 years [SD = 1.21; 14–18 years) from across Israel and instructed them to fill out a battery of questionnaires online. From the total sample, 31.4% classified themselves as secular, 24.7% classified themselves as traditional, 40.9% classified themselves as religious and 3.0% classified themselves as ultra-orthodox. As above, a dichotomous variable was created for religious affiliation by combining ‘Secular’ and ‘Traditional’ under the variable ‘low religious affiliation’ and ‘Religious’ and ‘Ultra-Orthodox’ as ‘high religious affiliation’.

#### 4.1.2. Self-Report Measures

##### Metacognitions

The Metacognitions Questionnaire 30 (MCQ-30; [51])) was used as in Study 1. In this study, Cronbach’s alpha was 0.88.

##### Impulsiveness

The Barratt Impulsiveness Scale-11 (BIS-11; [91] translated to Hebrew by Glicksohn, Leshem and Aharoni [92]) was used as in Study 1. In this study, Cronbach’s alpha was 0.82.

##### Thought Suppression

For this study, we adapted the Food Thought Suppression Inventory (FTSI; [94]) by translating the survey from English into Hebrew and then back-translating it into English. The translators were instructed to replace each reference to thoughts about food with a reference to thoughts about using social networks. The Thought Suppression Inventory is a 15-item unidimensional self-report measures that assesses the tendency to avoid thoughts related to social networks use (e.g., “There are images about social networks use that come to mind that I cannot erase”) using a 5-point Likert scale (1 = Strongly disagree and 4 = Strongly agree). Higher scores indicate higher levels of thought suppression. In this study, Cronbach’s alpha was 0.94.

##### Problematic Social Networks Use

PSNU was measured with the nine items of the Social Media Disorder Scale [116] that was first translated into Hebrew by a translator who was fluent in both languages and then back-translated into English by the author. These nine items measured the same nine criteria that were used to measure Internet gaming disorder, but then applied to social networks use, i.e., Tolerance, Withdrawal, Displacement, Escape, Problems, Deception, Displacement, and Conflict. Participants were asked to complete the sentence “During the past year, have you…” using a 5-point scale ranging from 1 = Never to 5 = Very often (e.g., “…tried to spend less time on social networks, but failed). Higher scores indicate higher levels of problematic social networks use. In this study, Cronbach’s alpha was 0.65.

### 4.2. Procedure

This was the same as in Studies 1 and 2.

### 4.3. Data Analysis

**Figure 4:**
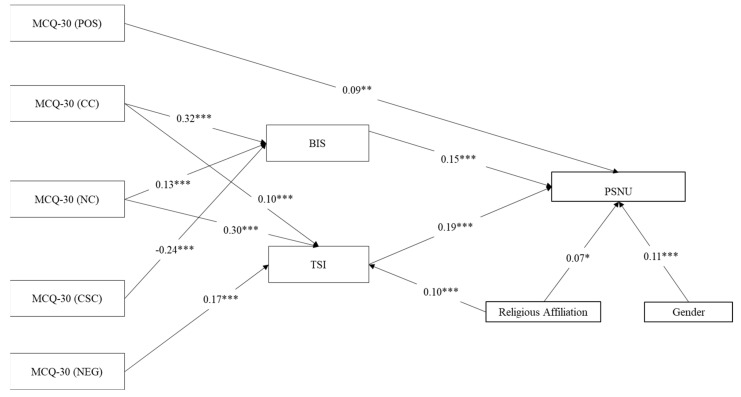
Results of the path analytical model with PSNU as outcome variable. Notes: * *p* < 0.05, ** *p* < 0.001, *** *p* < 0.001; *n* = 1004; Coefficients are standardized estimates; Religious Affiliation = (1 = Low, 2 = High); Gender: 1 = M, 2 = F; MCQ-30 (POS) = Metacognitions Questionnaire-30 (Positive Beliefs about Worry); MCQ-30 (NEG) = Metacognitions Questionnaire-30 (Negative Beliefs about Thoughts concerning Uncontrollability and Danger); MCQ-30 (CC) = Metacognitions Questionnaire-30 (Cognitive Confidence); MCQ-30 (NC) = Metacognitions Questionnaire-30 (Beliefs about the Need to Control Thoughts); MCQ-30 (CSC) = Metacognitions Questionnaire-30 (Cognitive Self-Consciousness); BIS = Barret Impulsivity Scale; TSI = Thought Suppression Inventory; PSNU = Problematic Social Networks Use.

Bivariate correlations among the variables were calculated using SPSS (version 25, IBM, New York, NY, USA; [99]. A series of Shapiro-Wilk normality tests indicated that all of the variables were non-normally distributed at the *p* < 0.001 level. Accordingly, all correlations were conducted using Spearman’s Rho (see Table 1). Then, the pattern of relationships specified by our theoretical model (Figure 1) was tested using path analyses with the same parameters as in Study 1. Specifically, in the tested models, PSNU was the outcome variable, impulsiveness and thought suppression were the mediators, and the five MCQ-30 metacognitions (i.e., positive beliefs about worry, negative beliefs about thoughts concerning uncontrollability and danger, cognitive confidence, beliefs about the need to control thoughts, and cognitive self-consciousness) were the independent variables, whereas age, gender, and religious affiliation (low vs. high) were included as control variables on the two mediators and the outcome (Figure 1). We first tested the full model in order to assess which model was the most plausible. Subsequently, path coefficients not significant at the 5% level were removed step-by-step. Figure 4 shows the final model including significant path coefficient only.

### 4.4. Results

#### 4.4.1. Bivariate Correlations

PSNU was moderately correlated with thought suppression (*r_s_* = 0.32, *p* < 0.001) and had a weak correlation with impulsiveness (*r_s_* = 0.21, *p* < 0.001), gender (*r_s_* = 0.13, *p* < 0.001), cognitive confidence (*r_s_* = 0.15, *p* < 0.001), positive beliefs about worry (*r_s_* = 0.15, *p* < 0.001), negative beliefs about thoughts concerning uncontrollability and danger (*r_s_* = 0.14, *p* < 0.001), beliefs about the need to control thoughts (*r_s_* = 0.12, *p* < 0.001) and religiosity (*r_s_* = 0.09, *p* < 0.001).

#### 4.4.2. Path Analysis: Do Thought Suppression and Impulsiveness Mediate the Association between Metacognitions and PSNU?

The model was run on a final sample of *n* = 1000 adolescents (as 3 participants of the total sample of *n* = 1003 did not complete one or more scale). Positive beliefs about worry were directly and positively associated with PSNU (though weakly). Cognitive confidence and beliefs about the need to control thoughts were positively associated with impulsiveness and thought suppression, with cognitive confidence showing the strongest association with impulsiveness and beliefs about the need to control thoughts showing the strongest association with thought suppression. Negative beliefs about thoughts concerning danger and uncontrollability were positively associated to thought suppression only, whereas cognitive self-consciousness was negatively associated to impulsiveness only. In turn, impulsiveness and thought suppression were both positively associated to PSNU (Figure 4). As regard the control variables, religious affiliation was positively associated with thought suppression and problematic social networks use, whereas gender had a positive association with PSNU.

The results of the Sobel test with respect to indirect relationships highlighted the mediating role of impulsiveness between: (i) cognitive confidence and PSNU (*β* = 0.048, SE = 0.035, *z* = 4.615, *p* < 0.001); (ii) beliefs about the need to control thoughts and PSNU (*β* = 0.019, SE = 0.027, *z* = 2.845, *p* = 0.004); and cognitive self-consciousness and PSNU (*β* = −0.035, SE = 0.031, *z* = −4.069, *p* < 0.001). Moreover, results supported the mediating role of thought suppression between: (i) negative beliefs about thoughts concerning uncontrollability and danger and PSNU (*β* = 0.032, SE = 0.027, *z* = 3.810, *p* < 0.001); (ii) cognitive confidence and PSNU (*β* = 0.019, SE = 0.023, *z* = 2.776, *p* = 0.005); and (iii) beliefs about the need to control thoughts and PSNU (*β* = 0.004, SE = 0.005, *z* = 2.729, *p* = 0.006).

When considering the model fit, the model was able to explain 10% of the variance of PSNU, 22% of the variance of thought suppression and 16% of the variance of impulsiveness. Overall, it can be said that the model was an acceptable fit to analyze the data in light of the total amount of variance that it explained (Total Coefficient of Determination, TCD = 0.36). In fact, such a TCD value corresponds to a correlation of *r* = 0.60, which can be reasonably described as a large effect size [107].

## 5. Discussion

Study 3 was designed to examine whether thought suppression and impulsiveness mediate the associations between metacognitions and PSNU. As in Study 2, we found that positive beliefs about worry were directly and positively associated with PSNU, which perfectly fits with the backfire effect that was found to be related to the process [110].

Moreover, as in Studies 1 and 2, similar mediation paths were revealed regarding impulsiveness. Specifically, we found that lack of confidence in one’s mnemonic and attentional capabilities, beliefs about the need to control thoughts, and lack of cognitive self-consciousness were associated with higher impulsiveness; higher impulsiveness, in turn, was linked with higher severity of PSNU. Study 3 also indicated that thought suppression mediated the associations between metacognitions and PSNU, although only two out of the three mediating paths replicated those of Study 2. As in Study 2, we found that lack of confidence in one’s mnemonic and attentional capabilities, and beliefs about the need to control thoughts were associated with higher thought suppression; higher social-networks-use-related thought suppression was linked, in turn, with higher severity of PSNU. Unlike Study 2, we found that negative beliefs about thoughts concerning uncontrollability and danger, and not lack of cognitive self-consciousness were linked to social-networks-use-related thought suppression. This discrepancy may stem from two factors: (i) In each study we used a topic-specific thought suppression measurement that might alter the pattern of associations; and (ii) In Study 2, we examined CSBD, which is more prevalent among boys and in Study 3 we examined PSNU, which is more prevalent among girls. Although no gender differences were reliably recorded in metacognitions (e.g., [51,117], research has indicated differences in thought suppression between genders such that women tend to use it more often than men (e.g., [118]). These differences may account for the differences in results of Studies 2 and 3.

### 5.1. General Summary Discussion

The goal of the current three-study research was to investigate the association between metacognitions, thought suppression, impulsiveness, and three behavioral addictions among adolescents (IGD, CSBD and PSNU). On the whole, the study’s findings highlight the relative significance of various metacognitions in the prediction of behavioral addictions as well as the mediating role of impulsiveness, partly thought suppression, between metacognitions and behavioral addictions.

The current research has revealed consistent and reliable mediation paths involving impulsiveness. Specifically, we found that the absence of cognitive confidence, the conviction that thoughts need to be controlled, and a low cognitive self-consciousness were all linked with higher impulsiveness and via high impulsiveness with all the behavioral addictions we examined.

The current research also revealed equivocal results regarding domain-specific thought suppression. Whereas metacognitions were not linked to thought suppression in IGD, lack of confidence in one’s thoughts or judgments, and beliefs about the need to control thoughts. were linked to thought suppression in CSBD and PSNU.

Two metacognitions therefore clearly emerge as ‘transdiagnostic’ factors in predicting, broadly, impulsiveness and thought suppression that are at the core of (most) behavioral addictions in adolescents: lack of cognitive confidence and beliefs about the need to control thoughts. Why would this be the case? Cognitive confidence refers to a subjective belief about the validity of one’s thoughts or judgments. The degree of confidence can vary from extreme certainty to extreme doubt in the validity of memories, decisions, and judgement. Cognitive confidence is important because it affects whether people translate their individual thoughts into more general judgments, and whether these judgments in turn are influential in guiding behavior. Here, we found that lack of cognitive confidence is tightly linked with both impulsiveness and thought suppression, which might reflect an inhibition in the translation of thoughts into concrete judgements and so more impulsive and less guided behaviors [35]. Beliefs about the need to control thoughts are likely to activate strategies (such as desire thinking, rumination and worry as well as thought suppression) which may make, paradoxically, thoughts become more salient in consciousness as well as increase in affective responses that are linked to experiencing such thoughts (e.g., a sexual or gaming urge). This, in turn, could bring to an escalation in the sense of deprivation for a given target (e.g., pornography, gaming, etc.) and greater impulsive behavior [35].

### 5.2. Therapeutic Implications of the Current Research

The current research has important implications for many clinical and health issues. First and foremost, the current research might help to better tailor psychotherapeutic interventions for adolescents with behavioral addictions. Over the last twenty-five years the Self-Regulatory Executive Function (S-REF) model has offered novel insights into the role of metacognition in psychopathology [47,48], and specifically to the development of a novel form of psychological therapy, Metacognitive Therapy (MCT; [119]). MCT was successfully employed as an intervention for various addictions such as alcohol use [120] and substance abuse [121]. From the metacognitive standpoint, psychological disturbances are maintained by the activation of the Cognitive-Attentional Syndrome (CAS). The CAS encompasses repetitive negative thinking styles (rumination and worry) as well as thought suppression and maladaptive self-monitoring. The activation of the CAS brings an increase of attentional focus toward a specific stimulus and a feedback loop that fail to regulate the related thoughts and behaviors. The activation, perseveration and escalation of the CAS is linked to the presence of unhelpful metacognitions. Using MCT techniques such as detached mindfulness might prove to be effective in reducing negative meta-appraisal of addiction-related thoughts (internet-gaming-related, sexual-related, and/or social-networks-related) and by doing so potentially reduce impulsive decision making and actions and thus the severity of the behavioral addictions. It is worthy to note, at this juncture, that MCT has not been extensively tested in young people hence its effectiveness in tackling psychological dysfunction in this group remains to be confirmed.

### 5.3. Limitations

Although our main hypotheses were supported, the research has several limitations. First, Studies 1 to 3 are correlational in nature, which makes it impossible to draw conclusions regarding potential causal processes between metacognitions and addictive behaviors via impulsiveness and thought suppression. Determining the direction of the associations among adolescents would be possible only with the help of longitudinal studies. Secondly, the sample consisted of Israeli, Jewish adolescents. Subsequent studies might test the generalizability of our model using more diverse populations.

## 6. Concluding Remarks

This is the first study to show, in an adolescent population, the role played by metacognitions in predicting common behavioral addictions (IGD, CSBD and PSNU) through both thought suppression and impulsiveness pathways. These findings broadly support a metacognitive a conceptualization of psychopathology as applied to behavioral addictions and showcase the importance of meta-belief systems and control strategies in younger populations.

## Figures and Tables

**Table 1:** Means, standard deviations and bivariate correlations with outcome variables across each study.

	IGD *n* = 471–474	CSBD *n* = 453	PSNU *n* = 1003
1. Gender			
Correlation	−0.26 **	−0.28 **	0.13 **
2. Age			
Correlation	−0.09 *	−0.09	0.05
3. Religious Affiliation			
Correlation	0.10 *	0.15 **	0.09 **
4. MCQ-30 (POS)			
Mean	12.14	12.55	12.17
Standard Deviation	3.97	4.04	3.99
Correlation	0.12 *	0.15 **	0.15 **
5. MCQ-30 (NEG)			
Mean	13.09	13.07	13.26
Standard Deviation	4.55	4.51	4.49
Correlation	0.15 **	0.14 **	0.15 **
6. MCQ-30 (CC)			
Mean	11.15	10.95	11.05
Standard Deviation	4.46	4.16	4.35
Correlation	0.22 **	0.24 **	0.15 **
7. MCQ-30 (NC)			
Mean	13.50	13.06	13.45
Standard Deviation	3.57	3.51	3.61
Correlation	0.17 **	0.28 **	0.13 **
8. MCQ-30 (CSC)			
Mean	16.95	16.70	16.72
Standard Deviation	3.86	4.11	4.03
Correlation	0.17 **	0.04	0.04
9. BIS			
Mean	65.67	66.84	67.33
Standard Deviation	10.05	10.55	11.24
Correlation	0.28 **	0.29 **	0.21 **
10. TSI			
Mean	26.48	37.13	32.83
Standard Deviation	12.70	14.97	14.31
Correlation	0.68 **	0.68 **	0.32 **

Note: Age = Age in Years; MCQ-30 (POS) = Metacognitions Questionnaire-30 (Positive Beliefs about Worry); MCQ-30 (NEG) = Metacognitions Questionnaire-30 (Negative Beliefs about Thoughts concerning Uncontrollability and Danger); MCQ-30 (CC) = Metacognitions Questionnaire-30 (Cognitive Confidence); MCQ-30 (NC) = Metacognitions Questionnaire-30 (Beliefs about the Need to Control Thoughts); MCQ-30 (CSC) = Metacognitions Questionnaire-30 (Cognitive Self-Consciousness); BIS = Barret. Impulsivity Scale; TSI = Thought Suppression Inventory; IGD = Internet Gaming Disorder; CSBD = Compulsive Sexual Behavior Disorder; PSNU = Problematic Social Networks Use. *n* 453 to 1003; * *p* < 0.05; ** *p* < 0.01.

## Data Availability

Data for this study can be obtained from the first author.

## References

[1] World Health Organization (2018). International Classification of Diseases for Mortality and Morbidity Statistics-Eleventh Revision (ICD-11).

[2] Yau M.Y.H., Potenza M.N. (2015). Gambling disorder and other behavioral addictions: Recognition and treatment. Harv. Rev. Psychiatry.

[3] Paulus F.W., Ohmann S., Von Gontard A., Popow C. (2018). Internet gaming disorder in children and adolescents: A systematic review. Dev. Med. Child Neurol..

[4] Efrati Y., Dannon P. (2018). Normative and clinical self-perceptions of sexuality and their links to psychopathology among adolescents. Psychopathology.

[5] Efrati Y., Gola M. (2018). Understanding and predicting profiles of compulsive sexual behavior among adolescents. J. Behav. Addict..

[6] Andreassen C.S. (2015). Online social network site addiction: A comprehensive review. Curr. Addict. Rep..

[7] Li J.B., Wu A.M., Feng L.F., Deng Y., Li J.H., Chen Y.X., Mo P.K.H., Lau J.T. (2020). Classification of probable online social networking addiction: A latent profile analysis from a large-scale survey among Chinese adolescents. J. Behav. Addict..

[8] Ryan T., Chester A., Reece J., Xenos S. (2014). The uses and abuses of Facebook: A review of Facebook addiction. J. Behav. Addict..

[9] Gola M., Potenza M.N. (2018). Promoting educational, classification, treatment, and policy initiatives: Commentary on: Compulsive sexual behaviour disorder in the ICD-11 (Kraus et al., 2018). J. Behav. Addict..

[10] Kafka M.P. (2010). Hypersexual disorder: A proposed diagnosis for DSM-V. Arch. Sex. Behav..

[11] World Health Organization (2018). ICD-11 (Mortality and Morbidity Statistics). https://icd.who.int/dev11/lm/en#/http://id.who.int/icd/entity/1630268048.

[12] Kraus S.W., Krueger R.B., Briken P., First M.B., Stein D.J., Kaplan M.S., Voon V., Abdo C.H.N., Grant J.E., Atalla E. (2018). Compulsive sexual behaviour disorder in the ICD-11. World Psychiatry.

[13] Kraus S.W., Voon V., Potenza M.N. (2016). Should compulsive sexual behavior be considered an addiction?. Addiction.

[14] Potenza M.N., Gola M., Voon V., Kor A., Kraus S.W. (2017). Is excessive sexual behaviour an addictive disorder?. Lancet Psychiatry.

[15] Miller W.R., Forcehimes A.A., Zweben A. (2019). Treating Addiction: A Guide for Professionals.

[16] Grant J.E., Potenza M.N., Weinstein A., Gorelick D.A. (2010). Introduction to behavioral addictions. Am. J. Drug Alcohol. Abus..

[17] Wines D. (1997). Exploring the applicability of criteria for substance dependence to sexual addiction. Sex. Addict. Compuls. J. Treat. Prev..

[18] Bőthe B., Tóth-Király I., Griffiths M.D., Potenza M.N., Orosz G., Demetrovics Z. (2021). Are sexual functioning problems associated with frequent pornography use and/or problematic pornography use? Results from a large community survey including males and females. Addict. Behav..

[19] Hormes J.M., Kearns B., Timko C.A. (2014). Craving F acebook? Behavioral addiction to online social networking and its association with emotion regulation deficits. Addictive.

[20] Kuss D.J., Griffiths M.D. (2011). Online social networking and addiction—A review of the psychological literature. Int. J. Environ. Res. Public Health.

[21] Wise K., Alhabash S., Park H. (2010). Emotional responses during social information seeking on Facebook. Cyberpsychol. Behav. Soc. Netw..

[22] Pontes H.M., Schivinski B., Sindermann C., Li M., Becker B., Zhou M., Montag C. (2019). Measurement and conceptualization of Gaming Disorder according to the World Health Organization framework: The development of the Gaming Disorder Test. Int. J. Ment. Health Addict..

[23] Wenzlaff R.M., Wegner D.M. (2000). Thought suppression. Annu. Rev. Psychol..

[24] Brockman R., Ciarrochi J., Parker P., Kashdan T. (2017). Emotion regulation strategies in daily life: Mindfulness, cognitive reappraisal and emotion suppression. Cogn. Behav. Ther..

[25] Gross J.J., John O.P. (2003). Individual differences in two emotion regulation processes: Implications for affect, relationships, and well-being. J. Personal. Soc. Psychol..

[26] Abramowitz J.S., Tolin D.F., Street G.P. (2001). Paradoxical effects of thought suppression: A meta-analysis of controlled studies. Clin. Psychol. Rev..

[27] Wenzlaff R.M., Luxton D.D. (2003). The role of thought suppression in depressive rumination. Cogn. Ther. Res..

[28] Efrati Y. (2019). God, I can’t stop thinking about sex! The rebound effect in unsuccessful suppression of sexual thoughts among religious adolescents. J. Sex. Res..

[29] Efrati Y., Kolubinski C.D., Caselli G., Spada M.M. (2020). Desire thinking as a predictor of compulsive sexual behaviour in adolescents: Evidence from a cross-cultural validation of the Hebrew version of the Desire Thinking Questionnaire. J. Behav. Addict..

[30] Gong X., Chen C., Lee M.K. (2020). What drives problematic online gaming? The role of IT identity, maladaptive cognitions, and maladaptive emotions. Comput. Hum. Behav..

[31] Klein A.A. (2007). Suppression-induced hyperaccessibility of thoughts in abstinent alcoholics: A preliminary investigation. Behav. Res. Ther..

[32] Erskine J.A., Ussher M., Cropley M., Elgindi A., Zaman M., Corlett B. (2012). Effect of thought suppression on desire to smoke and tobacco withdrawal symptoms. Psychopharmacology.

[33] Riley B. (2014). Experiential avoidance mediates the association between thought suppression and mindfulness with problem gambling. J. Gambl. Stud..

[34] Wang D.A., Hagger M.S., Chatzisarantis N.L.D. (2020). Ironic Effects of Thought Suppression: A Meta-Analysis. Perspect. Psychol. Sci..

[35] Spada M.M., Caselli G., Nikčević A.V., Wells A. (2015). Metacognition in addictive behaviors. Addict. Behav..

[36] Dalley J.W., Everitt B.J., Robbins T.W. (2011). Impulsivity, compulsivity, and top-down cognitive control. Neuron.

[37] Zhang Y., Qiu X., Ren Q., Zhou Z., Zhou H., Du J., Voo V., Zhang C., Liu W. (2020). Psychometric Properties of the Chinese version of UPPS-P Impulsive Behavior Scale. Front. Psychiatry.

[38] Bőthe B., Tóth-Király I., Potenza M.N., Griffiths M.D., Orosz G., Demetrovics Z. (2019). Revisiting the role of impulsivity and compulsivity in problematic sexual behaviors. J. Sex. Res..

[39] Wetterneck C.T., Burgess A.J., Short M.B., Smith A.H., Cervantes M.E. (2012). The role of sexual compulsivity, impulsivity, and experiential avoidance in internet pornography use. Psychol. Rec..

[40] Wegmann E., Müller S.M., Turel O., Brand M. (2020). Interactions of impulsivity, general executive functions, and specific inhibitory control explain symptoms of social-networks-use disorder: An experimental study. Sci. Rep..

[41] Fumero A., Marrero R.J., Bethencourt J.M., Peñate W. (2020). Risk factors of internet gaming disorder symptoms in Spanish adolescents. Comput. Hum. Behav..

[42] Castellani B., Rugle L. (1995). A comparison of pathological gamblers to alcoholics and cocaine misusers on impulsivity, sensation seeking, and craving. Int. J. Addict..

[43] Hartmann A., Zeeck A., Barrett M.S. (2010). Interpersonal problems in eating disorders. Int. J. Eat. Disord..

[44] Lee R.S., Hoppenbrouwers S., Franken I. (2019). A systematic meta-review of impulsivity and compulsivity in addictive behaviors. Neuropsychol. Rev..

[45] Efrati Y., Gola M. (2019). The effect of early life trauma on compulsive sexual behavior among members of a 12-step group. J. Sex. Med..

[46] Efrati Y., Shukron O., Epstein R. (2019). Compulsive sexual behavior and sexual offending: Differences in cognitive schemas, sensation seeking, and impulsivity. J. Behav. Addict..

[47] Wells A., Matthews G. (1996). Modelling cognition in emotional disorder: The S-REF model. Behav. Res. Ther..

[48] Wells A., Matthews G. (1994). Attention and emotion: A clinical perspective. Clin. Psycbol. Psychother.

[49] Spada M.M., Proctor D., Caselli G., Strodl E. (2013). Metacognition in substance misuse. Princ. Addict. Compr. Addict. Behav. Disord..

[50] Cartwright-Hatton S., Wells A. (1997). Beliefs about worry and intrusions: The Meta-Cognitions Questionnaire and its correlates. J. Anxiety Disord..

[51] Wells A., Cartwright-Hatton S. (2004). A short form of the metacognitions questionnaire: Properties of the MCQ-30. Behav. Res. Ther..

[52] Wells A. (2013). Advances in metacognitive therapy. Int. J. Cogn..

[53] Hamonniere T., Varescon I. (2018). Metacognitive beliefs in addictive behaviours: A systematic review. Addict. Behav..

[54] Akbari M. (2017). Metacognitions or distress intolerance: The mediating role in the relationship between emotional dysregulation and problematic internet use. Addict. Behav. Rep..

[55] Jauregui P., Urbiola I., Estevez A. (2016). Metacognition in pathological gambling and its relationship with anxious and depressive symptomatology. J. Gambl. Stud..

[56] Lindberg A., Fernie B.A., Spada M.M. (2011). Metacognitions in problem gambling. J. Gambl. Stud..

[57] Mansueto G., Pennelli M., De Palo V., Monacis L., Sinatra M., De Caro M.F. (2016). The role of metacognition in pathological gambling: A mediation model. J. Gambl. Stud..

[58] Mansueto G., Caselli G., Ruggiero G.M., Sassaroli S. (2019). Metacognitive beliefs and childhood adversities: An overview of the literature. Psychol. Health Med..

[59] Marino C., Vieno A., Moss A.C., Caselli G., Nikčević A.V., Spada M.M. (2016). Personality, motives and metacognitions as predictors of problematic Facebook use in university students. Personal. Individ. Differ..

[60] Moneta G.B. (2011). Metacognition, emotion, and alcohol dependence in college students: A moderated mediation model. Addict. Behav..

[61] Nikcevic A.V., Spada M.M. (2008). Metacognitions across the continuum of smoking dependence. Behav. Cogn. Psychother..

[62] Spada M.M., Mohiyeddini C., Wells A. (2008). Measuring metacognitions associated with emotional distress: Factor structure and predictive validity of the Metacognitions Questionnaire 30. Personal. Individ. Differ..

[63] Spada M.M., Marino C. (2017). Metacognitions and emotion regulation as predictors of problematic internet use in adolescents. Clin. Neuropsychiatry.

[64] Spada M.M., Nikčević A.V., Moneta G.B., Wells A. (2007). Metacognition as a mediator of the relationship between emotion and smoking dependence. Addict. Behav..

[65] Spada M.M., Roarty A. (2015). The relative contribution of metacognitions and attentional control to the severity of gambling in problem gamblers. Addict. Behav. Rep..

[66] Spada M.M., Wells A. (2005). Metacognitions, emotion and alcohol use. Clin. Psychol. Psycholther..

[67] Spada M.M., Zandvoort M., Wells A. (2007). Metacognitions in problem drinkers. Cogn. Res..

[68] Aydın O., Güçlü M., Ünal-Aydın P., Spada M.M. (2020). Metacognitions and emotion recognition in Internet Gaming Disorder among adolescents. Addict. Behav. Rep..

[69] Marino C., Marci T., Ferrante L., Altoè G., Vieno A., Simonelli A., Spada M.M. (2019). Attachment and problematic Facebook use in adolescents: The mediating role of metacognitions. J. Behav. Addict..

[70] Miller L.R., Walshe E.A., McIntosh C.W., Romer D., Winston F.K. (2021). “What Were They Thinking?”: Metacognition and Impulsivity Play a Role in Young Driver Risk-Taking. J. Psychiatry Behav. Sci..

[71] Gomes F.C., de Andrade A.G., Izbicki R., Almeida A.M., de Oliveira L.G. (2013). Religion as a protective factor against drug use among Brazilian university students: A national survey. Rev. Bras. Psiquiatr..

[72] Hodge D.R., Andereck K., Montoya H. (2007). The protective influence of spiritual-religious lifestyle profiles on tobacco use, alcohol use, and gambling. Soc. Work. Res..

[73] Montgomery B.E., Stewart K.E., Bryant K.J., Ounpraseuth S.T. (2014). Dimensions of religion, depression symptomatology, and substance use among rural African American cocaine users. J. Ethn. Subst. Abus..

[74] Faigin C.A., Pargament K.I., Abu-Raiya H. (2014). Spiritual struggles as a possible risk factor for addictive behaviors: An initial empirical investigation. Int. J. Psychol. Relig..

[75] Grubbs J.B., Perry S.L., Wilt J.A., Reid R.C. (2019). Pornography problems due to moral incongruence: An integrative model with a systematic review and meta-analysis. Arch. Sex. Behav..

[76] Grubbs J.B., Perry S.L. (2019). Moral incongruence and pornography use: A critical review and integration. J. Sex. Res..

[77] Efrati Y. (2018). Adolescent compulsive sexual behavior: Is it a unique psychological phenomenon?. J. Sex. Marital. Ther..

[78] Efrati Y. (2019). A response to Pirutinsky’s (2019) religion and compulsive sexuality. J. Sex Res..

[79] Grubbs J.B., Grant J.T. (2020). Spirituality/Religion and Behavioral Addictions. Handbook of Spirituality, Religion, and Mental Health.

[80] Braun B., Kornhuber J., Lenz B. (2016). Gaming and religion: The impact of spirituality and denomination. J. Relig. Health.

[81] Kowalewska E., Gola M., Kraus S.W., Lew-Starowicz M. (2020). Spotlight on Compulsive Sexual Behavior Disorder: A Systematic Review of Research on Women. Neuropsychaiatr. Dis. Treat..

[82] Peter J., Valkenburg P.M. (2016). Adolescents and pornography: A review of 20 years of research. J. Sex. Res..

[83] Efrati Y., Amichai-Hamburger Y. (2021). Adolescents who solely engage in online sexual experiences are at higher risk for compulsive sexual behavior. Addict. Behav..

[84] Griffiths M.D., Kuss J.D., King L.D. (2012). Video game addiction: Past, present and future. Curr. Psychol. Rev..

[85] Lee Y.S., Han D.H., Kim S.M., Renshaw P.F. (2013). Substance abuse precedes internet addiction. Addict. Beyond Behav..

[86] Şaşmaz T., Öner S., Kurt A.Ö., Yapıcı G., Yazıcı A.E., Buğdaycı R., Şiş M. (2014). Prevalence and risk factors of Internet addiction in high school students. Eur. J. Public Health.

[87] Sugaya N., Shirasaka T., Takahashi K., Kanda H. (2019). Bio-psychosocial factors of children and adolescents with internet gaming disorder: A systematic review. Biopsychosoc. Med..

[88] Festl R., Scharkow M., Quandt T. (2013). Problematic computer game use among adolescents, younger and older adults. Addiction.

[89] Mihara S., Higuchi S. (2017). Cross-sectional and longitudinal epidemiological studies of I nternet gaming disorder: A systematic review of the literature. Psychol. Clin. Neuropsychiatry.

[90] Peris M., de la Barrera U., Schoeps K., Montoya-Castilla I. (2020). Psychological risk factors that predict social networking and internet addiction in adolescents. Int. J. Environ. Res. Public Health.

[91] Patton J.H., Stanford M.S., Barratt E.S. (1995). Factor structure of the Barratt Impulsiveness Scale. J. Clin. Psychol..

[92] Glicksohn J., Leshem R., Aharoni R. (2006). Impulsivity and time estimation: Casting a net to catch a fish. Personal. Individ. Differ..

[93] Vasconcelos A.G., Malloy-Diniz L., Correa H. (2012). Systematic review of psychometric proprieties of Barratt Impulsiveness Scale Version 11 (BIS-11). Clin. Neuropsychiatry.

[94] Barnes R.D., Fisak B., Tantleff-Dunn S. (2009). Validation of the Food Thought Suppression Inventory. J. Health Psychol..

[95] Barnes R.D., White M.A. (2010). Psychometric properties of the Food Thought Suppression Inventory in men. J. Health Psychol..

[96] Pontes H.M., Griffiths M.D. (2015). Measuring DSM-5 Internet gaming disorder: Development and validation of a short psychometric scale. Comput. Hum. Behav..

[97] American Psychiatric Association (2013). Diagnostic and Statistical Manual of Mental Health Disorders.

[98] Qualtrics LLC Qualtrics XM. 2019, Qualtrics, Provo. https://www.qualtrics.com/au/research-core/survey-software/.

[99] IBM Corp. (2017). IBM SPSS Statistics for Windows.

[100] Rosseel Y. (2012). Lavaan: An R package for structural equation modeling. J. Stat. Softw..

[101] R Core Team (2013). R: A Language and Environment for Statistical Computing [Computer Software Manual].

[102] Satorra A., Bentler P.M., Von Eye A., Clogg C.C. (1994). Corrections to test statistics and standard errors in covariance structure analysis. Latent Variable Analysis. Applications for Developmental Research.

[103] Baron R.M., Kenny D.A. (1986). The moderator–mediator variable distinction in social psychological research: Conceptual, strategic, and statistical considerations. J. Personal. Soc. Psychol..

[104] Hayes A.F. (2009). Beyond Baron and Kenny: Statistical mediation analysis in the new millennium. Commun. Monogr..

[105] Bollen K.A. (1989). Structural Equations with Latent Variables.

[106] Jöreskog K.G., Sörbom D. (1996). LISREL 8: User’s Reference Guide.

[107] Cohen J. (1988). Statistical Power Analysis for Behavioral Science.

[108] Marino C., Spada M.M. (2017). Dysfunctional cognitions in online gaming and internet gaming disorder: A narrative review and new classification. Curr. Addict. Rep..

[109] Sun X., Zhu C., So S.H.W. (2017). Dysfunctional metacognition across psychopathologies: A meta-analytic review. Eur. Psychiatry.

[110] Wells A. (2000). Emotional Disorders and Metacognition: Innovative Cognitive Therapy.

[111] Efrati Y., Mikulincer M. (2018). Individual-based Compulsive Sexual Behavior Scale: Its development and importance in examining compulsive sexual behavior. J. Sex. Mar. Ther..

[112] Efrati Y., Gola M. (2019). Adolescents’ compulsive sexual behavior: The role of parental competence, parents’ psychopathology, and quality of parent–child communication about sex. J. Behav. Addict..

[113] Efrati Y., Gola M. (2018). Compulsive sexual behavior: A twelve-step therapeutic approach. J. Behav. Addict..

[114] Efrati Y., Gerber Z., Tolmacz R. (2019). The relation of intra-psychic and relational aspects of the self to compulsive sexual behavior. J. Sex. Marital. Ther..

[115] Efrati Y., Shukron O., Epstein R. (2020). Early Maladaptive Schemas Are Highly Indicative of Compulsive Sexual Behavior. Eval. Health Prof..

[116] Van Den Eijnden R.J., Lemmens J.S., Valkenburg P.M. (2016). The social media disorder scale. Comput. Hum. Behav..

[117] Kolić-Vehovec S., Bajšanski I., Zubković B.R. (2010). Metacognition and reading comprehension: Age and gender differences. Trends and Prospects in Metacognition Research.

[118] Robichaud M., Dugas M.J., Conway M. (2003). Gender differences in worry and associated cognitive-behavioral variables. J. Anxiety Disord..

[119] Wells A. (2009). Metacognitive Therapy for Anxiety and Depression.

[120] Caselli G., Martino F., Spada M.M., Wells A. (2018). Metacognitive therapy for alcohol use disorder: A systematic case series. Front. Psychol..

[121] Inchausti F., Ortuño-Sierra J., García-Poveda N.V., Ballesteros-Prados A. (2017). Metacognitive abilities in adults with substance abuse treated in therapeutic community Habilidades metacognitivas en adultos con abuso de sustancias bajo tratamiento en comunidad terapéutica. Adicciones.

